# Investigating the electronic and magnetic properties of Na_*x*_Fe_1/2_Mn_1/2_O_2_ cathode materials with X-ray Compton scattering

**DOI:** 10.1039/d5ra04468e

**Published:** 2026-07-02

**Authors:** Veenavee Nipunika Kothalawala, Kosuke Suzuki, Johannes Nokelainen, Ilja Makkonen, Erica West, Lassi Roininen, Jere Leinonen, Pekka Tynjälä, Petteri Laine, Juho Välikangas, Ulla Lassi, Assa Aravindh Sasikala Devi, Matti Alatalo, Yuki Mizuno, Naruki Tsuji, Hikaru Usami, Yuju Nagasaki, Tsuyoshi Takami, Yoshiharu Sakurai, Hiroshi Sakurai, Mohammad Babar, Venkatasubramanian Vishwanathan, Arun Bansil, Bernardo Barbiellini

**Affiliations:** a Department of Physics, School of Engineering Sciences, LUT University FI-53851 Lappeenranta Finland veenavee.kothalawala@lut.fi; b Graduate School of Science and Technology, Gunma University Kiryu Gunma 376-8515 Japan; c Department of Mechanical Engineering, School of Energy Systems, LUT University FI-53851 Lappeenranta Finland; d Department of Physics, Northeastern University Boston Massachusetts 02115 USA; e Quantum Materials and Sensing Institute, Northeastern University Burlington MA 01803 USA; f Department of Physics, University of Helsinki, FI-00014 University of Helsinki P.O. Box 43tbox43 Helsinki Finland; g Department of Computational Engineering, School of Engineering Sciences, LUT University FI-53851 Lappeenranta Finland; h Research Unit of Sustainable Chemistry, University of Oulu Oulu Finland; i Kokkola University Consortium Chydenius, University of Jyväskylä Kokkola Finland; j Materials and Mechanical Engineering Research Unit, University of Oulu Oulu Finland; k Nano and Molecular Systems Research Unit, University of Oulu Pentti Kaiteran Katu 1 90570 Oulu Finland; l Japan Synchrotron Radiation Research Institute (JASRI) Sayo Hyogo 679-5198 Japan; m Otemon Gakuin University 2-1-15 Nishiai Ibaraki Osaka 567-8502 Japan; n College of Engineering, Aerospace Engineering, University of Michigan Ann Arbor MI 48109 USA

## Abstract

We discuss the electronic and magnetic properties of Na_*x*_Fe_1/2_Mn_1/2_O_2_, a promising Na-ion battery cathode material. Using X-ray Compton scattering, SQUID magnetometry, and density-functional-theory-based modeling, we probe how electrons and spins evolve during sodiation. By comparing the Compton profiles of sodiated and desodiated samples, we show that oxygen 2p orbitals drive the redox process, while transition-metal 3d electrons become more delocalized, explaining the metallic phase at *x* = 2/3. These profile differences define a quantitative descriptor for the sodiation range associated with improved conductivity. Electron holes on oxygen, reflected in the oxygen magnetization, confirm the important role of oxygen in the electrochemical activity of the cathode.

## Abstract 2

We discuss the electronic and magnetic properties of Na_*x*_Fe_1/2_Mn_1/2_O_2_, a promising Na-ion battery cathode material. Using X-ray Compton scattering, SQUID magnetometry, and DFT-based modeling, we probe how electrons and spins evolve during sodiation.

## Graphical abstract

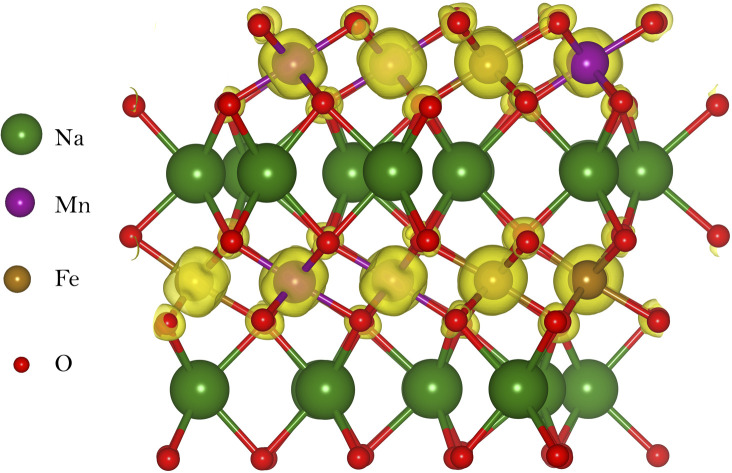

## 1 Introduction

The uneven geographical distribution of lithium (Li) reserves has significant societal impacts^1,2^ in terms of the sustainability of lithium-ion batteries for grid-scale energy storage systems. As a result, sodium-ion batteries (NIBs) have been gaining increasing attention due to their lower cost and the greater abundance of sodium (Na). The first commercially available NIBs were offered by Asian manufacturers and have been the subject of extensive analyses.^3^ Nevertheless, NIBs face challenges due to the larger atomic radius of Na compared to Li ions, resulting in slower Na^+^ diffusion kinetics that leads to a lower energy density and shorter cycle life.

The performance of NIBs is primarily influenced by the choice of cathode material. Among the various candidate materials, layered oxides, particularly NaMnO_2_, have shown great potential for applications.^4,5^ NaMnO_2_-based materials can be synthesized in different structural phases, such as O2, P2, O3 and P3, depending on the arrangement of the oxide layers and the sodium environment;^6^ here, ‘O’ denotes an octahedral environment for Na ions, while ‘P’ indicates a prismatic environment, and the number in O2, P2, O3 and P3 refers to the number of distinct interlayers involved in various oxide layer packings. Phase transitions between these structures can occur during the charging process, leading to cathode degradation.

**Fig. 1:**
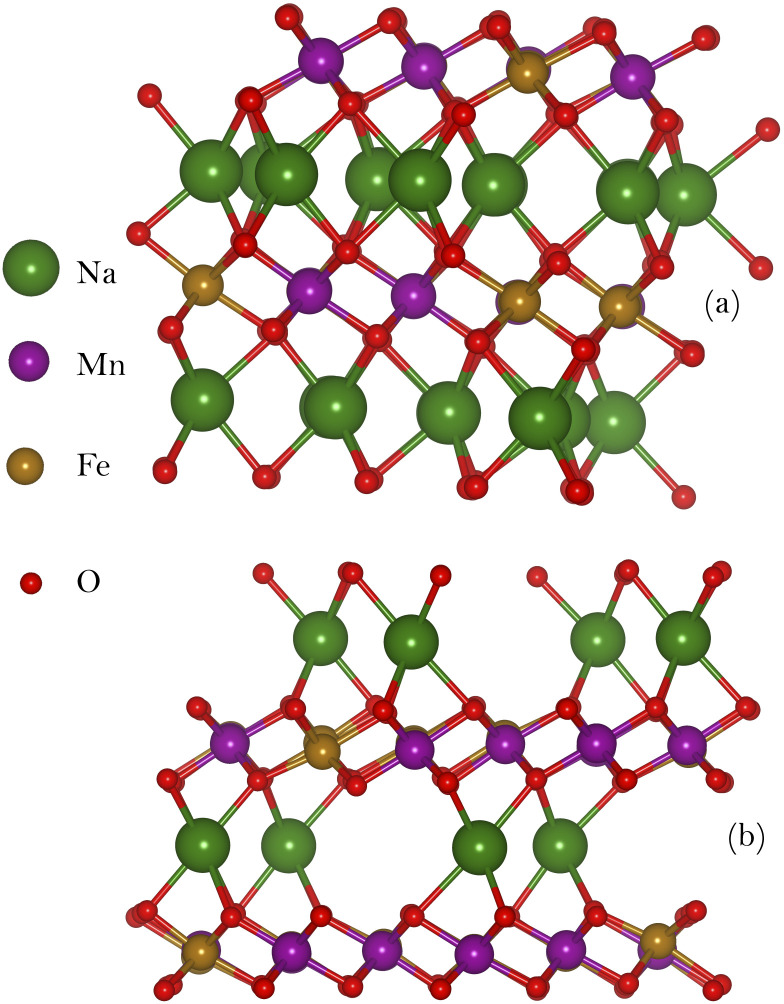
Structures of (a) P2-Na_2/3_Fe_1/2_Mn_1/2_O_2_ and (b) O2-Na_2/9_Fe_1/2_Mn_1/2_O_2_ used in our DFT calculations.

Recent studies have focused on addressing these challenges by substituting transition metals in the cathode structure. For example, doping with iron (Fe) has been shown to mitigate structural degradation and improve cycling performance. This strategy has led to the development of novel sodium-ion electrode materials, such as P2-Na_*x*_Fe_1/2_Mn_1/2_O_2_, which exhibits promising reversible capacity and is stable between *x* ∼ 0.4 and *x* = 2/3 at voltages as high as 3.8 V.^7^ However, when charged to 4.2 V, the diffraction patterns indicate a phase transition from the P2 to the O2 phase, which can lead to performance degradation.^7^ The P2 and O2 structures are illustrated in Fig. 1. Interestingly, Tang *et al.*^8^ recently suggested that this phase transition issue could be addressed by introducing vacancies into the transition-metal layer. Moreover, Wang *et al.*^9^ showed that oxygen vacancies (OVs) play a crucial role in enhancing Mn redox activity by lowering the Mn valence state. OVs, which are formed intrinsically or by anionic redox during high-voltage operation, also promote reversible Fe^3+^ migration to stabilize the structure and support redox reactions involving the oxygen sublattice. This dynamic interplay strengthens charge compensation and improves electrochemical performance. Overall, controlled OV engineering helps mitigate Mn^3+^ Jahn–Teller distortion and supports the development of stable, high-capacity Fe,Mn layered cathodes for NIBs.

To facilitate further advances, a comprehensive, atomic-level understanding of the fundamental processes underlying the redox reactions in NIBs is necessary. In this way, rational strategies for optimizing the performance and durability of sodium-based batteries can be designed.

Here, we use both regular and magnetic Compton X-ray scattering experiments combined with first-principles density functional theory (DFT) modeling to investigate the layered oxide cathode material Na_*x*_Fe_1/2_Mn_1/2_O_2_. By measuring the Compton profiles at sodium concentrations of 
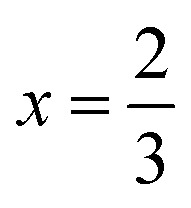
 and 
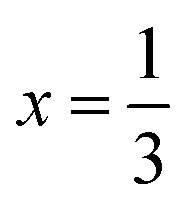
, we extract detailed information about the redox orbitals involved in the battery's operation. Moreover, we identify the magnetic orbitals associated with these two sodium concentrations by analyzing the associated magnetic Compton profiles. We emphasize that (non-magnetic) difference Compton profiles and magnetic Compton profiles yield complementary insights into the redox and magnetic orbitals, respectively.^10^

While Compton scattering has historically been viewed as a specialized technique, its scope has broadened significantly over the years. It is a nondestructive bulk-sensitive probe that measures the ground-state electronic momentum density of materials. Unlike conventional spectroscopies that are surface-sensitive or rely on optical transitions, Compton scattering directly probes the spatial distribution of valence electrons in momentum space. This enables detection of the changes in orbital occupancy and electron delocalization that accompany redox processes or magnetic ordering. In the context of battery materials, X-ray Compton scattering provides unique insights into the spatial extent and nature of redox orbitals, distinguishing between localized and delocalized characters, and thereby informing mechanisms of phase stability, capacity retention, and degradation, as demonstrated in LiCoO_2_ cathode materials.^11,12^ A recent review by Zuo *et al.*^13^ shows that Compton scattering can be applied to characterize commercial batteries under *operando* conditions, providing valuable information on Li-ion flow, degradation pathways, and structural changes during cycling. These capabilities position Compton scattering as a complementary tool to the more conventional spectroscopic and diffraction methods, and as a way of identifying spectroscopic descriptors that provide atomic-scale insight into the redox mechanisms underlying the operation of Li-ion batteries.^14,15^ Given the current interest in electrochemical energy storage, the integration of Compton scattering into this domain can be expected to provide new opportunities to connect fundamental atomic-level understanding of materials with macroscopic device performance.

## 2 Methods

### 2.1 Samples

Fe_1/2_Mn_1/2_CO_3_ precursors were synthesized *via* carbonate coprecipitation in an inert nitrogen atmosphere to prevent oxidation. The coprecipitation was carried out in a stirred-tank reactor using an overhead stirrer and a heating plate. An aqueous solution of metal sulfates (2 M FeSO_4_ + MnSO_4_) and 2 M Na_2_CO_3_ was fed into the reactor using peristaltic pumps. The feed rate of the metal solution was kept constant, while the pH was maintained at approximately 8 by adjusting the feed rate of the Na_2_CO_3_ solution. The temperature inside the reactor was held at 50 °C throughout the 6 h coprecipitation process. Similar coprecipitation conditions have been reported to strongly influence particle size distribution and precursor properties in Fe/Mn carbonate systems.^16^

After coprecipitation, the precursor slurry was vacuum-filtered, and the precipitate was thoroughly washed with deionized water. The resulting Fe_1/2_Mn_1/2_CO_3_ precursors were dried overnight at 60 °C in a vacuum oven. The particle size distribution of the precursor powder, measured *via* laser diffraction, yielded characteristic diameters of *D*_1_ = 6.75 µm, *D*_10_ = 8.11 µm, *D*_50_ = 10.6 µm, *D*_90_ = 13.6 µm, and *D*_99_ = 15.9 µm. The dried precursor material was subsequently milled and sieved under dry-room conditions. It was mixed with anhydrous Na_2_CO_3_ (99.5%, Alfa Aesar) using an IKA A11 basic mixer with two mixing cycles of 30 s separated by a cooling interval. Prior to mixing, the Na_2_CO_3_ was ground and sieved using a mortar and pestle. The powder mixtures were placed in alumina crucibles and calcined at 900 °C for 12 h under air, using a heating ramp of 2.5°C min^−1^. Molar ratios of Na(Fe_0.5_Mn_0.5_CO_3_) = 2/3 and 1/3 were used to synthesize Na_2/3_Fe_1/2_Mn_1/2_O_2_ and Na_1/3_Fe_1/2_Mn_1/2_O_2_, respectively. The small amount of sodium impurity present in the precursors was taken into account during weighing. After cooling, the calcined products were ground using a mortar and pestle and sieved through a 25 µm aperture sieve.

The crystal structures of the synthesized materials were determined *via* X-ray diffraction (XRD) using a SmartLab diffractometer (Rigaku Corporation) with CuK_α_ radiation (see the SI). The sample with *x* = 2/3 crystallizes in the P2 phase. The *x* = 1/3 sample also adopts the P2 phase, despite being located at the lower boundary of the reported P2 phase stability range (*x* ∼ 0.4 to *x* = 2/3),^7^ although it exhibits additional weak diffraction peaks indicative of minor impurity phases, as shown in Fig. S2 of the SI.

Magnetization measurements were performed using a SQUID magnetometer (MPMS-7, Quantum Design, Inc). Measurements were conducted at 10 K by sweeping the magnetic field from −2.5 T to 2.5 T.

### 2.2 DFT-based modeling

Our DFT calculations were based on the projector-augmented-wave method^17^ as implemented in the Vienna *ab initio* simulation package.^18,19^ To account for strong electron correlation effects, we used the regularized strongly-constrained-and-appropriately-normed r^2^SCAN^20^ exchange-correlation functional, which avoids the use of effective Hubbard parameters.^21^ We employed a plane-wave kinetic energy cut-off of 480 eV, which we found to provide a reasonable balance between computational cost and accuracy. This choice is supported by a previous study on a related sodium-ion battery material by Lee *et al.*,^22^ where convergence within approximately 3 meV per formula unit was achieved using a 450 eV cut-off, indicating that this value is adequate for the comparative purposes of our study. Gaussian smearing with a width of 0.05 eV (full width at half maximum) and a total energy tolerance of 10^−6^ eV were used to determine the self-consistent charge density. The Na (2p, 3s), Fe (3d, 4s), Mn (3p, 3d, 4 s), and O (2s, 2p) electrons were treated as valence electrons.

We based our DFT computations on atomic coordinates for the P2 (*x* = 2/3) and O2 (*x* = 2/9) phase models obtained by Zarrabeitia *et al.* in their detailed structural work,^23^ where the Fe and Mn cations are randomly distributed within the transition-metal layers in a 1 : 1 ratio; see Fig. 1. The P2-type structure corresponds to the sodiated phase formed at low voltage and the O2-type structure corresponds to the desodiated phase formed at high voltage. We adopted a ferromagnetic high-spin configuration for Fe and Mn following Zarrabeitia *et al.* A 4 × 4 × 1 (4 × 2 × 1) *k*-mesh was used to sample the Brillouin zone of P2 (O2). We relaxed these structures with an atomic force tolerance of 0.01 eV Å^−1^ and obtained the lattice constants of *a* = 2.913 Å, *c* = 11.113 Å for the P2 and *a* = 2.864 Å, *c* = 11.208 Å for the O2 phase. The P2-phase values are close to the experimental values of *a* = 2.9405 Å and *c* = 11.1957 Å.^24^ Bader charge analysis^25,26^ was used to compute atomic charges based on the DFT calculations.

### 2.3 Momentum density calculations

As already pointed out above, Compton scattering with X-rays^27,28^ has proven particularly useful in the advanced characterization of batteries.^12,15^ In a Compton scattering experiment, the electron momentum density *ρ*(**p**) is accessed *via* the Compton profile *J*(*p*_z_), which is derived from the Doppler broadening of the scattered photons. This analysis relies on the impulse approximation, which assumes that the binding energy of the electrons can be neglected. As a result, the Compton profile directly reflects the electronic momentum distribution, containing information about both localized and delocalized occupied orbitals. Kaplan *et al.*.^29^ discuss the underlying many-body formalism based on Dyson orbitals. The impulse approximation holds well for high-energy (≥100 keV) X-ray photons. The Compton profile *J*(*p*_*z*_) is given by:^29^

1 *J*(*p*_*z*_) = ∫∫*ρ*(*p*)d*p*_*x*_d*p*_*y*_,

where *p* = (*p*_*x*_, *p*_*y*_, *p*_*z*_) is the electron momentum, and *ρ*(*p*) is the electron momentum density, which, following eqn (18) in the Encyclopedia of Condensed Matter Physics,^30^ can be expressed as a sum over electronic orbitals as:2
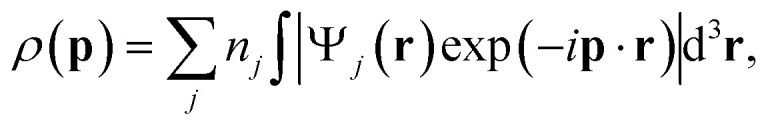
where *Ψ*_*j*_ is a natural spin orbital and *n*_*j*_ is the associated occupation number. Electron momentum density *ρ*(**p**) is often approximated by replacing the natural spin orbitals with the atomic Hartree-Fock orbitals^31^ and Kohn–Sham Bloch orbitals.^28^ Within the independent particle model, *n*_*j*_ = 1 if the state is occupied and *n*_*j*_ = 0 otherwise. The integral of *J*(*p*) gives the total number of electrons.

The magnetic Compton profile *J*_mag_(*p*_z_) is given by^30^

3 *J*_mag_(*p*_z_) = ∫∫(*ρ*_↑_(**p**)−*ρ*_↓_(**p**))d*p*_*x*_d*p*_*y*_,

where *ρ*_↑_(**p**) and *ρ*_↓_(**p**) are the momentum densities of the majority and minority spins, respectively. The spin magnetic moment *µ*_spin_ is obtained by integrating *J*_mag_(*p*_*z*_). The *z*-direction (*p*_*z*_) here corresponds to the scattering vector, which is aligned with the photon momentum transfer direction. In our experimental geometry, this nearly coincides with the incident photon propagation axis. The magnetic field was applied along the same axis. Since our samples are polycrystalline with randomly oriented domains, the measured spin-up and spin-down momentum densities both become spherically averaged. To obtain spherical averages theoretically, we use a Monte Carlo sampling approach coupled with linear interpolation. Since our study focuses on spherically-averaged spectra, we will hereafter replace *p*_*z*_ with the radial distance *p* = ‖**p**‖.

Usually, contributions to the Compton profiles from core orbitals^32^ are taken from the tabulated Compton profiles obtained within the Hartree–Fock scheme.^31^ Our theoretical Compton profiles for the valence electrons are based on our DFT computations following the method of Makkonen *et al.*^33^

### 2.4 Compton profile measurements

**Fig. 2:**
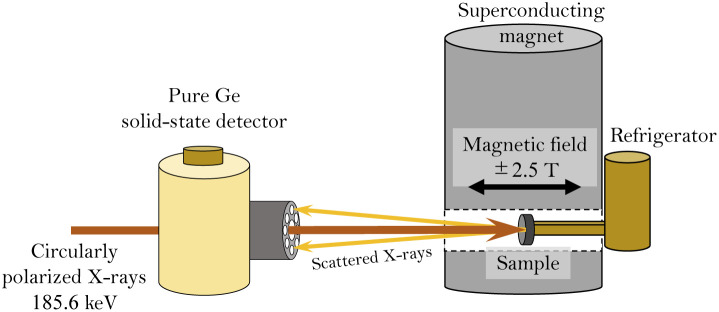
A schematic of the experimental magnetic Compton scattering setup at BL08W of SPring-8. The scattering geometry and orientation of the magnetic field are shown. The scattering vector lies along the *z*-direction of the Compton profile, which is nearly parallel to the incident photon direction, corresponding to a scattering angle of 178°. Momentum densities are spherically averaged in polycrystalline samples, while the spin remains aligned with the magnetic field.

Non-magnetic and magnetic Compton profiles were measured at the high-energy inelastic scattering beamline 08W at the Japanese synchrotron facility SPring-8.^34,35^ The experimental setup is illustrated in Fig. 2. The sample was irradiated with circularly polarized X-rays of 182.6 keV emitted from an elliptical multipole wiggler. The size of the incident X-ray beam at the sample position was 1 mm^2^. Compton scattered X-rays were measured using a pure Ge solid-state detector. The scattering angle was fixed at 178°. For the magnetic Compton profiles, a magnetic field of ± 2.5 T was applied to the sample that was kept at 7 K to obtain Compton scattered X-ray intensities, I+ and I−, by flipping the magnetic field every 60 s. Magnetic and non-magnetic Compton scattering experiments were both performed at 7 K.

## 3 Results and discussion

Bader charge analysis reveals that during the desodiation transition from the P2 (Na_2/3_Fe_1/2_Mn_1/2_O_2_) to the O2 (Na_2/9_Fe_1/2_Mn_1/2_O_2_) phase, charge transfer mostly occurs at the oxygen sites. Bader charges associated with oxygen atoms are on average 0.158*e* higher in the P2 compared to the O2 phase. In contrast, the corresponding relative Bader charges on the transition metals are 0.131*e* for Fe and 0.022*e* for Mn. 80.5% of the additional charge on the Fe_1/2_Mn_1/2_O_2_ planes in the P2 phase compared to the O2 phase is distributed on the oxygen sites. Sodium atoms, which act primarily as charge compensators, exhibit only minimal variation in charge (0.020*e*). Bader analysis thus supports the conclusion that oxygen anions play the dominant role in charge compensation upon desodiation from P2 to O2.

**Fig. 3:**
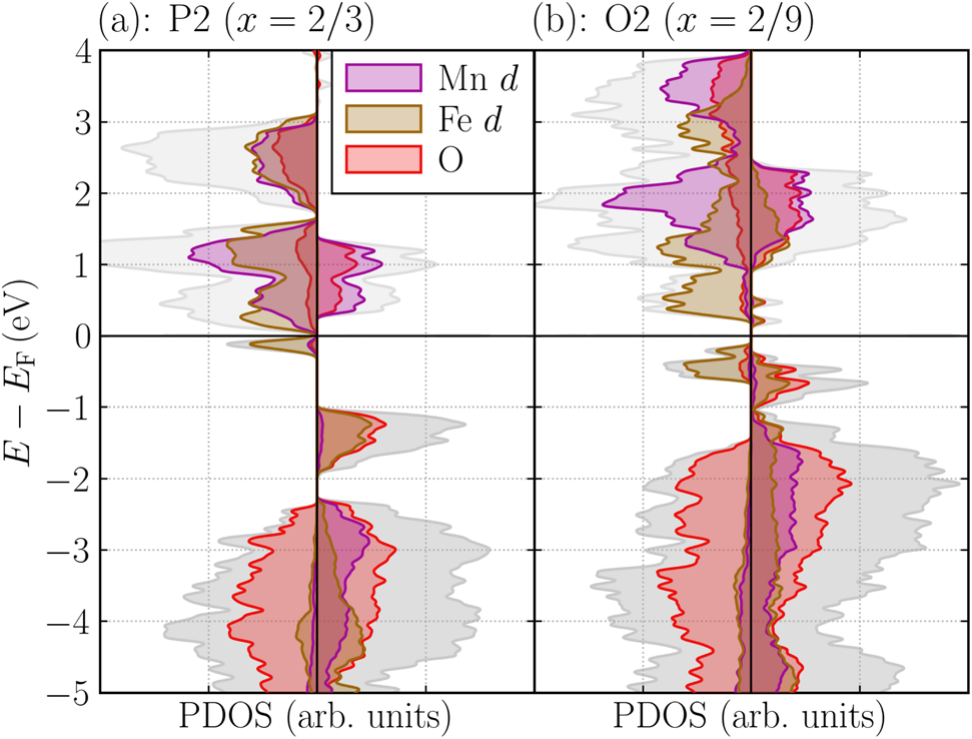
Spin- and orbital-resolved total and partial densities of states on various atomic sites (see legend) in (a) P2-Na_2/3_Fe_1/2_Mn_1/2_O_2_ and (b) O2-Na_2/9_Fe_1/2_Mn_1/2_O_2_.

The spin-resolved partial density of states (PDOS) for P2-Na_2/3_Fe_1/2_Mn_1/2_O_2_ and O2-Na_2/9_Fe_1/2_Mn_1/2_O_2_ is shown in Fig. 3. Our PDOS for the P2-type structure does not exhibit a band gap between the valence and conduction bands, but we observe a band gap for the O2-type structure. This is in contrast to the results reported by Zarrabeitia *et al.*,^23^ who used the PBE exchange–correlation functional^36^ with Hubbard corrections^21^ applied on the TM ions, and observed a band gap for P2 but not for O2. Our results, however, are consistent with the measured electronic resistivity values, which show that the sodium-ion-extracted electrode possesses a more insulating character compared to the sodiated sample.^23^ P2 PDOS results have also been reported by Abate *et al.*^37^ and Kim *et al.*^38^ The DFT calculations of Abate *et al.* do not display a gap near the Fermi energy, in agreement with our findings. This is likely because these authors included a Hubbard correction on the oxygen atoms. A consistent feature across all available PDOS results is the strong O-2p character near the Fermi level, indicating a substantial oxygen contribution to the redox orbitals.

**Fig. 4:**
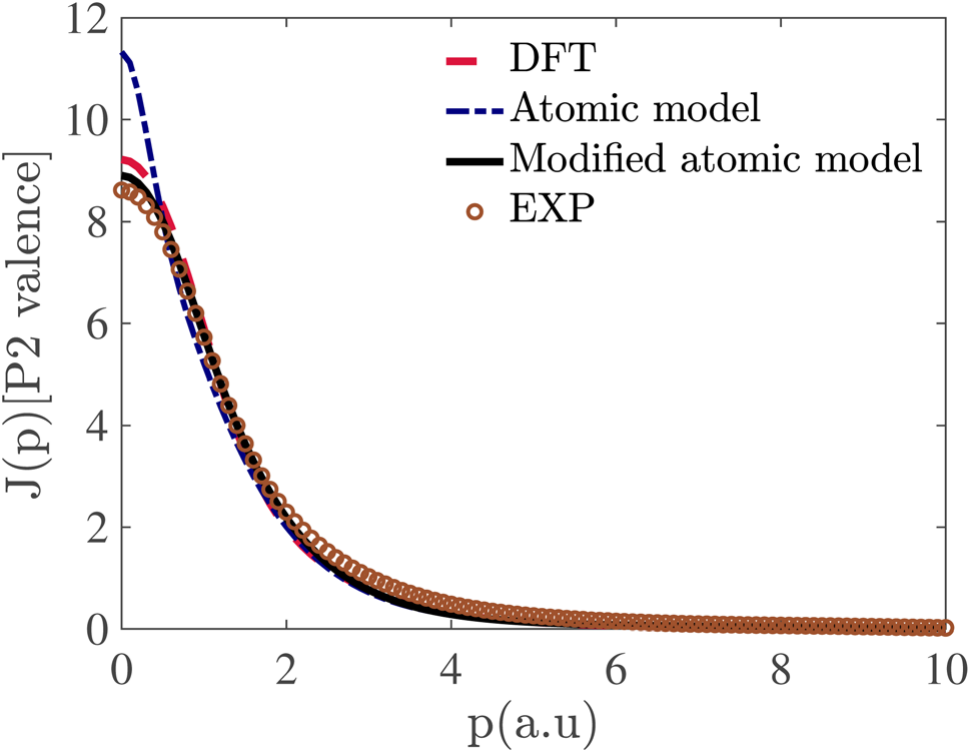
Spherically averaged theoretical and experimental valence Compton profiles of P_2_-Na_2/3_Fe_1/2_Mn_1/2_O_2_. Results for three different theoretical models are given (see text for details). Theoretical profiles are convoluted with a Gaussian of 0.5 a.u. full-width-at-half-maximum.

The experimental and computed valence Compton profiles of Na_2/3_Fe_1/2_Mn_1/2_O_2_ are presented in Fig. 4. We consider three different theoretical models: (1) an atomic orbital model;^32^ (2) a modified atomic orbital model,^39,40^ in which all positive ions donate electrons to the O-2p orbitals; and (3) first-principles DFT-based results for the valence electrons. The atomic orbital model is seen to be in poor agreement with the experimental profile, but the two other models are in better agreement. These results indicate that the electronic orbitals underlying these two models correctly capture the overall electron momentum distribution of this ionic layered oxide, and that the Na-3s electron here is mostly donated to the O-2p orbital.

Note that the modified atomic fit is consistent with ideal stoichiometry within the sensitivity of Compton scattering. In particular, oxygen vacancies at the level of several percent or higher would lead to a measurable redistribution of valence electrons, leaving excess charge on the Na-3s states. This would produce a clear and systematic mismatch between the experimental and calculated Compton profiles, most prominently in the low-momentum region, which is highly sensitive to the characteristic momentum distributions of O-2p and Na-3s orbitals.^41^ The absence of such deviations therefore rules out a significant (at about the percent-level^40^) concentration of defect vacancies in the present samples. An analysis along these lines for Na_1/3_Fe_1/2_Mn_1/2_O_2_ is presented in the SI (see Fig. S3).

The possible presence of oxygen vacancies is frequently discussed in the literature based on X-ray photoelectron spectroscopy (XPS) (see, *e.g.*, Su *et al.*,^42^ Su *et al.*,^43^ and Jiang *et al.*^44^). However, XPS is intrinsically surface sensitive, and O 1s peak deconvolution is non-unique and strongly affected by surface reconstruction and adsorbates in layered transition-metal oxides. X-ray absorption spectroscopy (XAS) provides a bulk-sensitive probe of O 2p-transition-metal 3d hybridization. In our sample characterization, we reproduce the characteristic lineshape reported by Abate *et al.*^37^ ( See Fig. S7 in the SI of Abate *et al.*^37^) for a defect-free sample, and we observe no features indicative of oxygen-deficient states. Compton scattering offers an independent and quantitative constraint on stoichiometry. Because the Compton profile directly measures the bulk electron momentum density normalized to the total number of electrons, oxygen vacancies would lead to a measurable reduction of O 2p electrons and a redistribution of valence electron momentum, particularly in the low-momentum region dominated by O 2p and Na 3s contributions. The excellent agreement between measured and calculated profiles, without systematic deviations, constrains deviations from ideal stoichiometry to below 1–2%, effectively ruling out any significant concentration of oxygen vacancies within the sensitivity of our measurements.

**Fig. 5:**
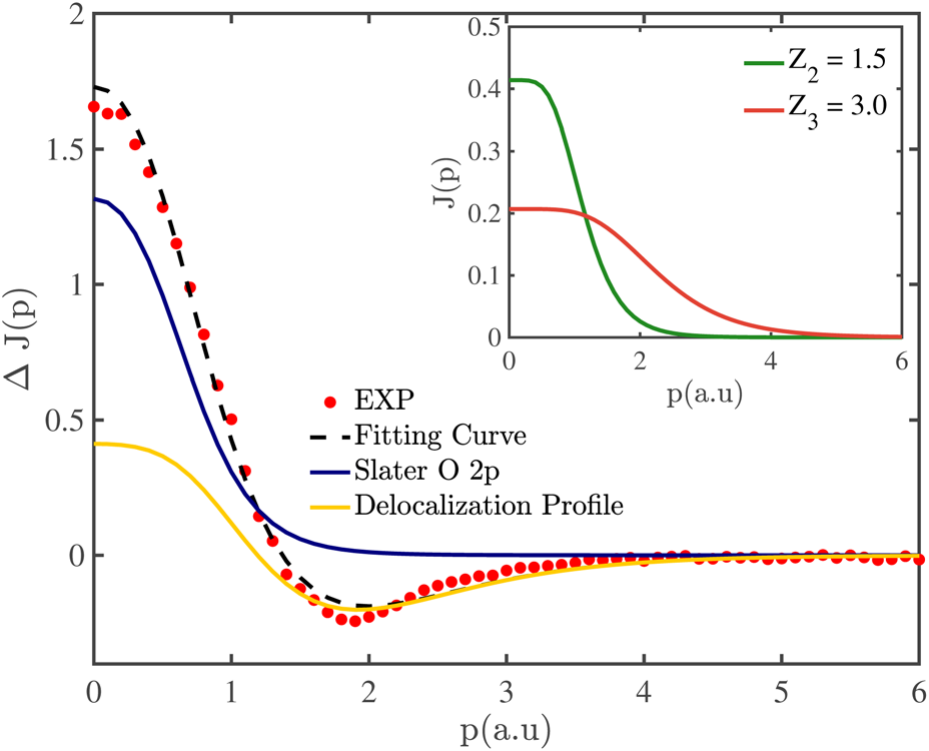
Experimental valence Compton profile difference Δ*J*(*p*) between P_2_-Na_2/3_Fe_1/2_Mn_1/2_O_2_ and O_2_-Na_1/3_Fe_1/2_Mn_1/2_O_2_, along with the corresponding curve-fitting results (see text for details), including contributions from O-2p and Fe/Mn 3d electrons. Inset shows the Compton profiles of transition-metal 3d orbitals for the indicated values of the *Z*_2_ and *Z*_3_ parameters. The profile *D*(*p*) is obtained by taking the difference between the profiles for *Z*_2_ = 1.5 and *Z*_3_ = 3. The area under the negative excursion of the *D*(*p*) profile quantifies the number of 3d electrons displaced during the sodiation process.

To investigate the redox mechanism in detail, we adopt a method previously applied successfully to Li-ion battery materials.^10–12,15,45,46^ Specifically, we analyze the redox activity by subtracting the valence Compton profile of Na_2/3_Fe_1/2_Mn_1/2_O_2_ from that of Na_1/3_Fe_1/2_Mn_1/2_O_2_, thereby isolating the redox-induced changes in the electronic structure. This difference profile, denoted as Δ*J*(*p*), is shown in Fig. 5 and it is modeled using Slater-type orbitals,^10^ which capture key physical properties, such as the correct exponential decay of electron density with distance, given by

4 *ψ*(*r*) ∝ *r*^*n*−1^*e*^−*Zr*^,

and a finite value at the nucleus. The spherically-averaged Compton profile for an O-2p orbital described by a Slater-type orbital is given by5
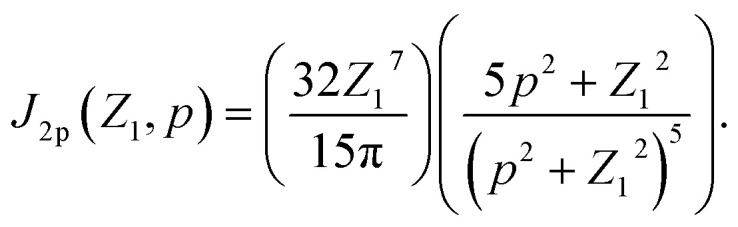


This orbital represents the primary destination for redox electrons donated by sodium. The spherical average of the Compton profile for a 3d shell, modeled using cubic harmonics, can be expressed as6
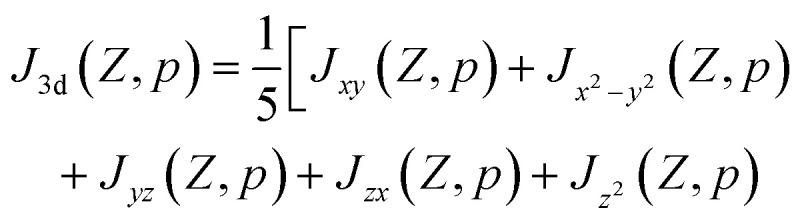
with the components given by:7
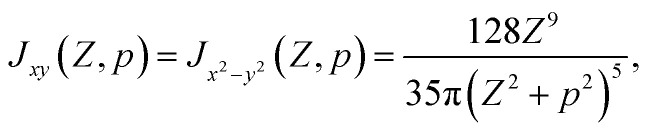
8
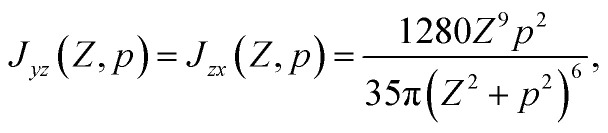
9
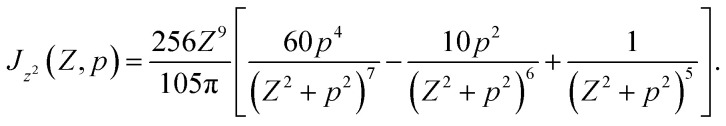


To investigate the redox process, we express the difference in valence Compton profiles between the two samples with different sodium concentrations as

10 Δ*J*(*p*) = *J*_2p_(*Z*_1_, *p*) + *D*(*p*),

where *J*_2p_(*Z*_1_, *p*) models the contribution of the electron transferred to the O 2p orbital. *D*(*p*) captures effects of reshuffling among the 3d orbitals without a net occupancy change, and is given by:

11 *D*(*p*) = *J*_3d_(*Z*_2_,*p*) − *J*_3d_(*Z*_3_,*p*),

The best fit yields *Z*_1_ = 1, *Z*_2_ = 1.5, and *Z*_3_ = 3. To quantify the number of displaced 3d electrons, we integrate the absolute value of the redistribution term:12
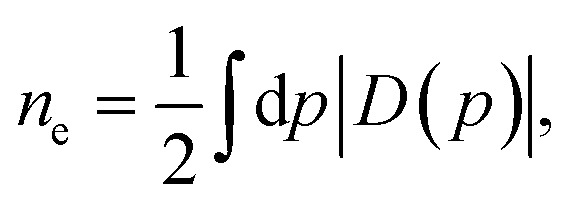
obtaining *n*_e_ = 0.17 electrons per Na atom. This quantitative insight—particularly the appearance of the negative excursion in Δ*J*(*p*)—provides a useful metric for tracking 3d electron delocalization and redox reversibility,^11^ which are key factors governing the performance of battery cathodes.^11^

**Fig. 6:**
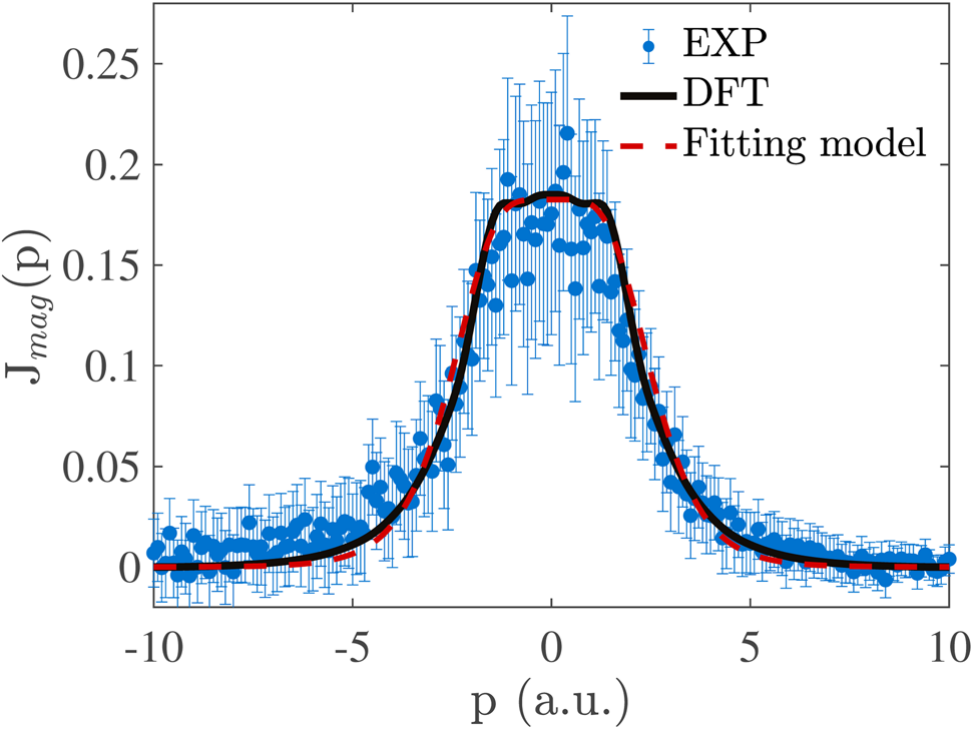
Experimental magnetic Compton profile of Na_2/3_Fe_1/2_Mn_1/2_O_2_ compared with the corresponding DFT results and two model fits discussed in the text.

**Fig. 7:**
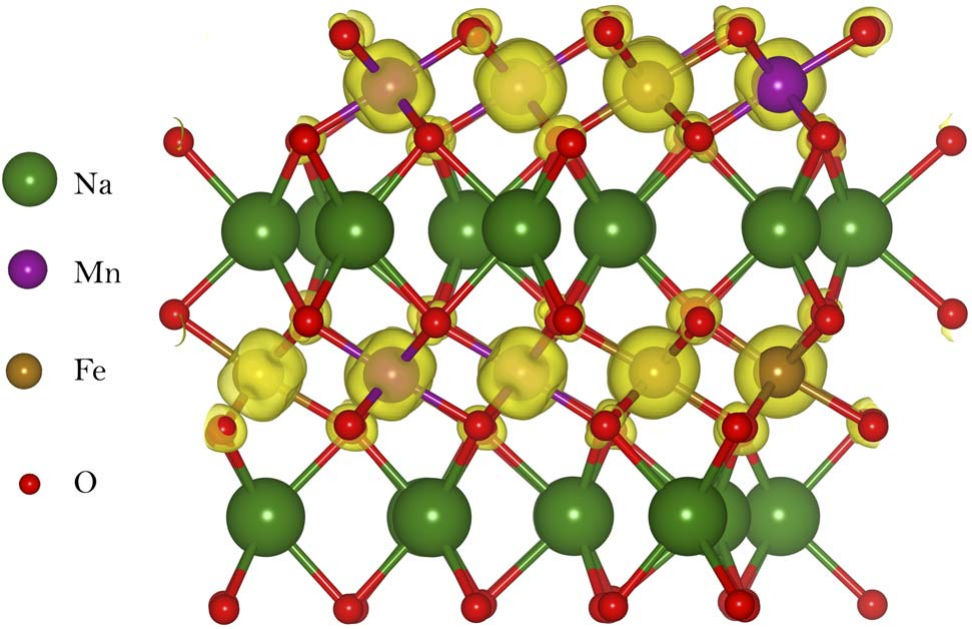
DFT-based spin density in Na_2/3_Fe_1/2_Mn_1/2_O_2_ with an isosurface value of 0.02 a.u.

The measured magnetic Compton profile *J*_mag_ shown in Fig. 6 indicates that our sample of Na_*x*_Fe_1/2_Mn_1/2_O_2_ has a net magnetization at *x* = 2/3. Interestingly, the DFT-based magnetic Compton profile for the same concentration exhibits remarkable agreement in shape to the measured *x* = 2/3 profile when both profiles are normalized to the magnetic moment, as shown in Fig. 6. To rationalize this shape, we use the sum of two Slater terms: a contribution from a transition-metal 3d Slater orbital with exponent *Z*_TM_ = 3.7 and another contribution from an O-2p orbital with exponent *Z*_O_ = 2. This fit yields a large O-2p contribution of about 5%, obtained by using Chi-square optimization applied to both the experimental noisy curve and the noise-free DFT profile. This magnetic oxygen contribution to *J*_mag_ is consistent with the DFT-based magnetization density distribution for *x* = 2/3, which is shown in Fig. 7. Even though an O^2−^ ion in isolation has a closed 2p^6^ shell and no net magnetic moment, in the cathode solid-state environment electron holes appear on the oxygen ions, changing their electronic and magnetic behavior. Interestingly, in our DFT calculations, a significant magnetization is observed from the oxygen ions in Na_2/3_Fe_1/2_Mn_1/2_O_2_ with values reaching 0.2 *µ*_B_ per O atom.

The magnetic Compton scattering experiment measures a magnetic moment of 0.45 *µ*_B_ per formula unit for *x* = 1/3 and 0.365 *µ*_B_ per formula unit for *x* = 2/3. In comparison, our DFT calculations, which adapt to the ferromagnetic high-spin configuration for Fe and Mn of Zarrabeitia *et al.*,^23^ predict much larger spin moments per formula unit: 3.833 *µ*_B_ for the P2 and 3.167 *µ*_B_ for the O2 phase. Note, however, that Compton scattering measurements capture the total magnetization of the system. Previous studies^38^ show that the magnetic layers of transition metals are ferromagnetic at low sodium content but become antiferromagnetic with increasing sodium content. This implies that the 2.5 T magnetic field used in our experiments is not strong enough to fully align the spins, which would explain why the measured magnetic moments are smaller than the corresponding theoretical values. Furthermore, we find that with the r2SCAN functional, it is possible to stabilize ferrimagnetic states with considerably lower total magnetization. However, an investigation of these interesting effects is out of the scope of this study.

**Fig. 8:**
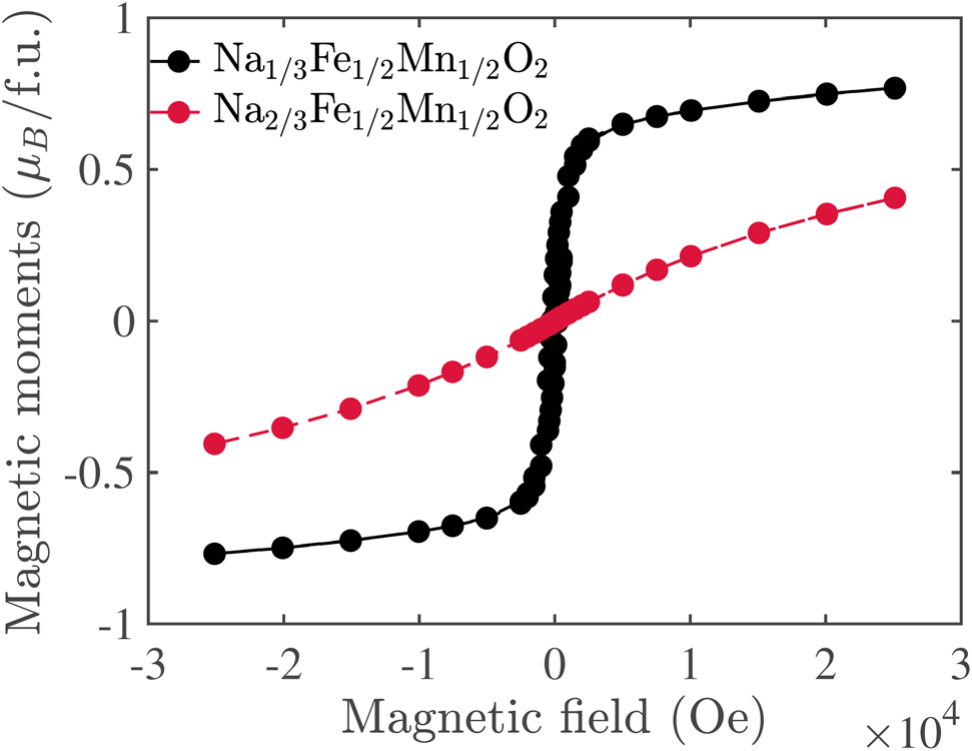
Magnetization curves obtained *via* SQUID experiments for samples with *x* = 1/3 and *x* = 2/3. Measurements were performed at low temperature *T* = 10 K. Magnetic field was scanned from −2.5 *T* to 2.5 *T*.

Fig. 8 shows the results of our SQUID measurements. This technique determines the total magnetic moment, which includes both orbital and spin contributions, whereas magnetic Compton scattering probes only the spin moment.^47^ For the *x* = 2/3 composition, the total measured magnetic moment is 0.406 *µ*_B_. In contrast, the total measured magnetic moment of the *x* = 1/3 sample is 0.768 *µ*_B_, which is significantly larger than the spin magnetic moment measured *via* the magnetic Compton scattering experiment. This indicates that the orbital magnetic contribution becomes substantial for *x* = 1/3. Finally, our results for *x* = 2/3 yield magnetization consistent with SQUID studies by Xu *et al.*^48^

## 4 Conclusion and outlook

We have investigated Na–Fe–Mn–O layered-oxide cathodes for sodium-ion batteries. This stable layered structure enables efficient Na-ion insertion and extraction, delivering promising cycling performance.^7^ Composed of abundant and eco-friendly elements, this material represents a cost-effective and sustainable alternative to lithium iron phosphate cathodes. Our study demonstrates that X-ray Compton scattering provides a sensitive, bulk-sensitive probe of the electronic structure in Na-ion battery cathodes. Combined total and magnetic Compton scattering measurements, supported by DFT-based momentum-space modeling, provide a detailed orbital and spin-resolved picture of redox activity in Na_*x*_Fe_1/2_Mn_1/2_O_2_ for *x* = 1/3 and *x* = 2/3. We identify oxygen 2p states as key contributors to the redox process.

Our analysis reveals the presence of a pronounced negative excursion in the sodiated difference Compton profile Δ*J*(*p*) at higher momenta, reflecting the delocalization of transition-metal 3d electrons. This feature, which is associated with electronic conductivity, serves as a descriptor for the sodiated metallic state (*x* = 2/3), and it could guide the optimization of reversible electrochemical devices, where low oxide conductivity limits efficiency. This descriptor is most relevant when O-2p orbitals dominate the redox process, accompanied by a partial delocalization of the transition-metal 3d electrons. We quantified electron transfer between the orbitals and found that extracting Na ions redistributes the 3d electrons, leading to an increased hole density in the O-2p orbital. This hole density, in turn, gives rise to the magnetization of the oxygen ion. It would be interesting to independently probe oxygen magnetization *via* magnetic circular dichroism experiments at the oxygen K-edge.^49^

We emphasize that our DFT r2SCAN calculations do not require any ad hoc Hubbard parameter, making our analysis particularly robust for modeling correlated systems. Moreover, our study demonstrates the value of Compton scattering in gaining an atomic-level understanding of the functional electronic states that govern battery behavior and confirms that Compton scattering is an excellent tool to detect light elements like oxygen.^50^ This approach provides a promising route for monitoring and optimizing charge transport and redox activity in Na-ion cathodes.

## Author contributions

Synthesis of sodium-ion precursor: Jere Leinonen and Petteri Laine. Synthesis of sodium-ion cathode material: Jere Leinonen, Juho Välikangas and Petteri Laine. Characterization of sodium-ion cathode material: Jere Leinonen, Juho Välikangas. DFT calculations: Veenavee Nipunika Kothalawala, Johannes Nokelainen, Ilja Makkonen. Modelling and theory: Erica West, Lassi Roininen, Assa Aravindh Sasikala, Matti Alatalo, Mohammad Babar, Venkatasubramanian Vishwanathan, Arun Bansil, and Bernardo Barbiellini. Visualization: Veenavee Nipunika Kothalawala, Johannes Nokelainen. Experiments: Kosuke Suzuki, Yuki Mizuno, Naruki Tsuji, Hikaru Usami, Yuju Nagasaki, Tsuyoshi Takami, Yoshiharu Sakurai, Hiroshi Sakurai. Writing – reviewing manuscript: Veenavee Nipunika Kothalawala, Bernardo Barbiellini, Johannes Nokelainen, Kosuke Suzuki, Jere Leinonen, Pekka Tynjälä, Ulla Lassi and Arun Bansil. Funding acquisition: Ulla Lassi, Venkatasubramanian Vishwanathan, Arun Bansil, Hiroshi Sakurai, Kosuke Suzuki and Bernardo Barbiellini.

## Conflicts of interest

The authors declare no competing interests.

## Supplementary Material

RA-016-D5RA04468E-s001

## Data Availability

The data that support the findings of this study are openly available in the GitHub repository at https://github.com/Veenavi92/NFMO_r2SCAN Supplementary information (SI) is available. See DOI: https://doi.org/10.1039/d5ra04468e.
