# Supplementary material for: Investigating the electronic and magnetic properties of NaxFe1/2Mn1/2O2 cathode materials with X-ray Compton scattering
Source: RSC Adv. 2026 Jul 2;16(34):32321–9. doi: 10.1039/d5ra04468e (PMC13325547; doi:10.1039/d5ra04468e)
Supplement: RA-016-D5RA04468E-s001 [file RA-016-D5RA04468E-s001.pdf]

Supplementary Information (SI) for RSC Advances.
This journal is © The Royal Society of Chemistry 2026

Supplemental material: Investigating the Electronic and Magnetic Properties of $Na_xFe_{1/2}Mn_{1/2}O_2$ Cathode Materials with x-ray Compton Scattering

Veenavee Nipunika Kothalawala[a,*], Kosuke Suzuki[b], Johannes Nokelainen[a,c,d,e], Ilja Makkonen[f], Erica West[g], Lassi Roininen[g], Jere Leinonen[h,i], Pekka Tynjälä[h,i], Petteri Laine[h,i], Juho Välikangas[h,i], Ulla Lassi[h,i], Assa Aravindh Sasikala Devi[h,j,*], Matti Alatalo[k], Yuki Mizuno[l], Naruki Tsuji[l], Hikaru Usami[b], Yuju Nagasaki[b], Tsuyoshi Takami[m], Yoshiharu Sakurai[l], Hiroshi Sakurai[b], Mohammad Babar[n], Venkat Vishwanathan[n], Arun Bansil[d,e], and Bernardo Barbiellini[a,d,e]

[a] *Department of Physics, School of Engineering Sciences, LUT University, FI-53851 Lappeenranta, Finland*
[b] *Graduate School of Science and Technology, Gunma University, Kiryu, Gunma 376-8515, Japan*
[c] *Department of Mechanical Engineering, School of Energy Systems, LUT University, FI-53851 Lappeenranta, Finland*
[d] *Department of Physics, Northeastern University, Boston, Massachusetts 02115, USA*
[e] *Quantum Materials and Sensing Institute, Northeastern University, Burlington, MA 01803, USA*
[f] *Department of Physics, University of Helsinki, P.O. Box 43, FI-00014 University of Helsinki, Helsinki, Finland*
[g] *Department of Physics, Northeastern University, Boston, Massachusetts 02115, USA*
[h] *Research Unit of Sustainable Chemistry, University of Oulu, Oulu, Finland*
[i] *Kokkola University Consortium Chydenius, University of Jyväskylä, Kokkola, Finland*
[j] *Materials and Mechanical Engineering Research Unit,University of Oulu, Oulu, Finland*
[k] *Nano and Molecular Systems Research Unit, University of Oulu, Pentti Kaiteran Katu 1, 90570 Oulu, Finland*
[l] *Japan Synchrotron Radiation Research Institute (JASRI), Sayo, Hyogo 679-5198, Japan*
[m] *Otemon Gakuin University, 2-1-15 Nishiai, Ibaraki, Osaka 567-8502, Japan*
[n] *College of Engineering, Aerospace Engineering, University of Michigan, Ann Arbor, MI 48109 USA*
[*] Veenavee.kothalawala@lut.fi, Assa.Sasikaladevi@oulu.fi

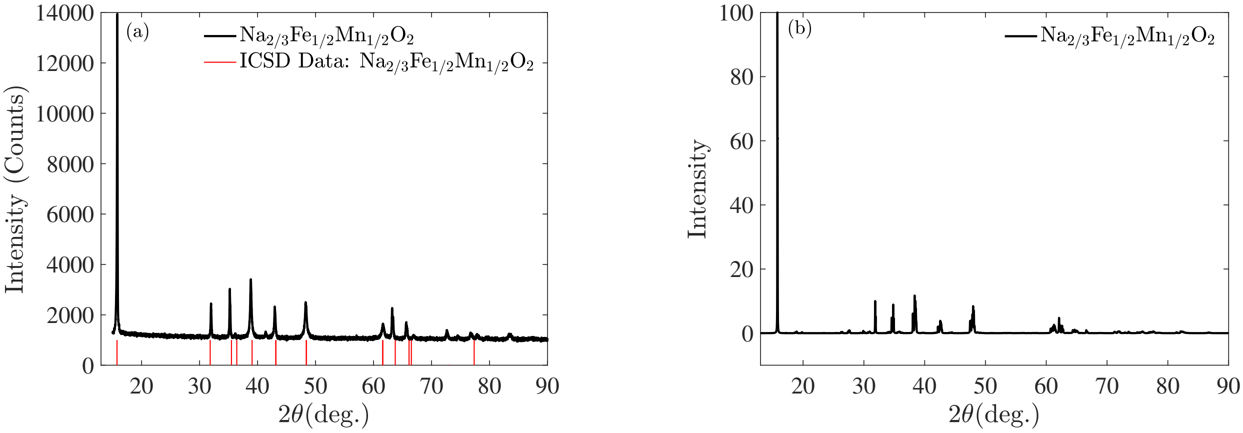

Figure S1: x-ray diffraction (XRD) patterns at the CuK_α fluorescence of $Na_{2/3}Fe_{1/2}Mn_{1/2}O_2$. (a) Experimental data. (b) Calculated pattern generated using VESTA software on the P2 phase model.

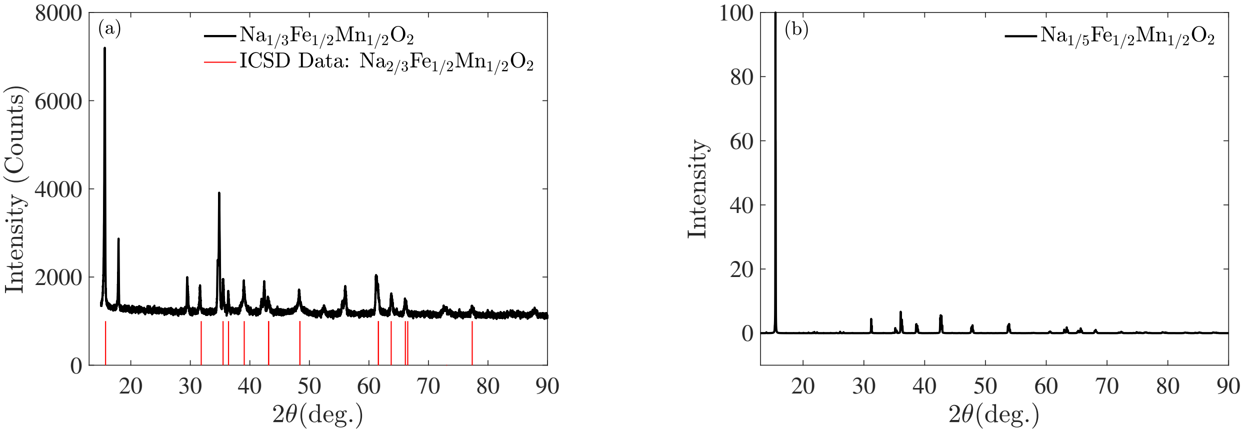

Figure S2: XRD patterns at the CuK_α fluorescence of $Na_{1/3}Fe_{1/2}Mn_{1/2}O_2$. (a) Experimental data showing a P2 phase contaminated by a secondary phase, which likely originates from incomplete formation of the layered structure under sodium-deficient conditions, leaving residual Na-poor oxides or related intermediate phases. (b) Calculated pattern generated using VESTA software on the O2 phase model for $Na_{1/5}Fe_{1/2}Mn_{1/2}O_2$.

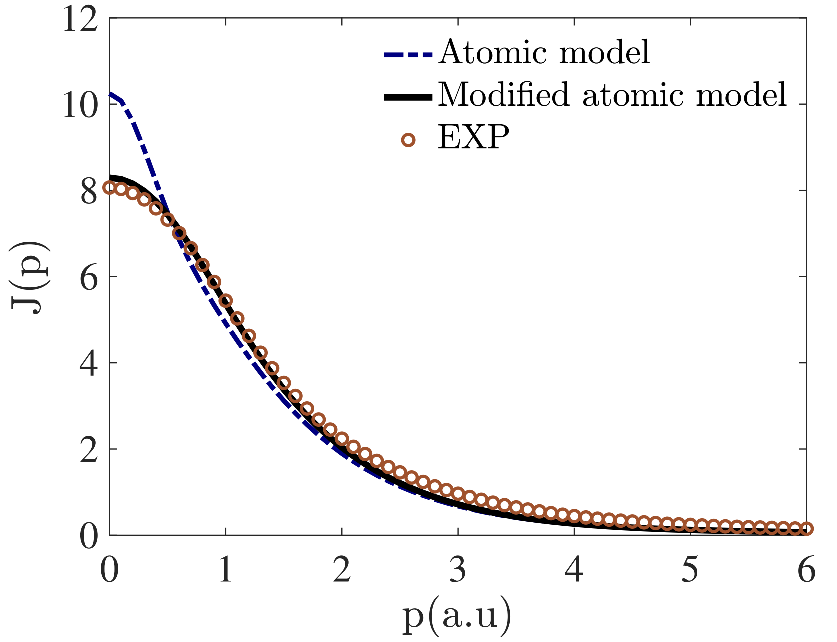

Figure S3: Spherically-averaged theoretical and experimental valence Compton profiles of $Na_{1/3}Fe_{1/2}Mn_{1/2}O_2$. Theoretical profiles are convoluted with a Gaussian of 0.5 a.u. full-width-at-half-maximum.

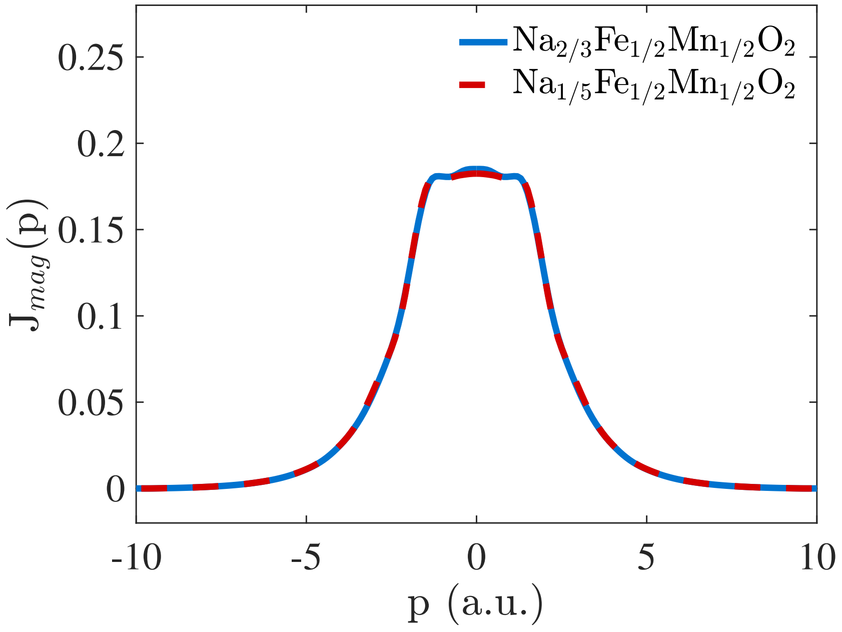

Figure S4: Spherically-averaged DFT based magnetic Compton profiles for $Na_{2/3}Fe_{1/2}Mn_{1/2}O_2$, and $Na_{2/9}Fe_{1/2}Mn_{1/2}O_2$. Two profiles are remarkably similar in that they can be collapsed onto a single curve when normalized to the same (unit) area.

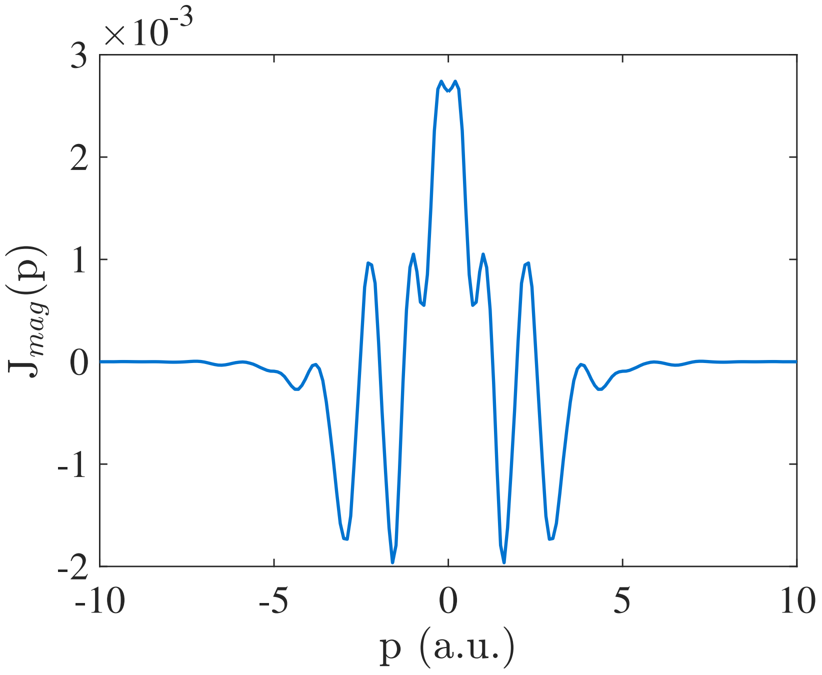

Figure S5: Difference between the spherically-averaged magnetic Compton profile of $Na_{2/3}Fe_{1/2}Mn_{1/2}O_2$ minus the same profile for $Na_{2/9}Fe_{1/2}Mn_{1/2}O_2$ based on DFT computations, where both profiles are normalized to the same (unit) area.

S1 Supplemental Data

All the data from the r2SCAN are provided at the Github repository: https://github.com/Veenavi92/NFMO_r2SCAN
